# The safety of fermented milk as a feedback method to reduce diarrhoea in newborn piglets

**DOI:** 10.17221/83/2025-VETMED

**Published:** 2026-01-26

**Authors:** Jan Matiasovic, Monika Zouharova, Petra Strakova, Lenka Kavanova, Daniela Karasova, Jan Gebauer, Arpad Csorgo, Ivan Rychlik

**Affiliations:** ^1^Veterinary Research Institute, Brno, Czech Republic; ^2^ARVET, Gabčíkovo, Slovak republic

**Keywords:** *Clostridium perfringens*, controlled oral exposure, sow

## Abstract

Diarrhoea in newborn piglets represents a significant challenge to pig production. Controlled oral exposure, also known as “feedback”, whereby sows are exposed at least two weeks before farrowing to pathogens that cause health problems in piglets, is a traditional method of diarrhoea prevention. One type of feedback involves fermenting cow’s milk with faeces from piglets suffering from diarrhoea and administering it to sows before farrowing. The bacterial composition of the faecal inoculum and fermented milk was compared in this study, and the safety of administering the fermented milk to pregnant sows was evaluated. Using microbiota characterisation by 16S rRNA gene sequencing, the genera *Acetobacter*, *Lactobacillus* and *Lactococcus* formed the core microbiota of the fermented milk. However, *Clostridium perfringens* accounted for up to 33% of the total microbiota in some fermented milk samples. Interestingly, the drop in pH during the later stages of fermentation inactivated *C. perfringens* and the samples were thus enriched for inactivated *C. perfringens* antigen. Our findings contribute to a better understanding of the mode of action of fermented milk when used as a form of feedback.

Diarrhoea in newborn piglets poses a substantial challenge to the global swine industry, resulting in significant economic losses through elevated morbidity and mortality rates, diminished growth performance, and escalating treatment costs (Sjolund et al. 2014; Schulz and Tonsor 2015). Neonatal piglet diarrhoea is a complex, multifactorial disease developing from the interplay of infectious agents, the host’s immunological competence, and various environmental and management factors (Racewicz et al. 2021; Fabiano and da Silva 2023). Key pathogens include a variety of viruses and bacteria, such as rotavirus, porcine epidemic diarrhoea (PED) virus, *Escherichia coli* and *Clostridium perfringens* (Vidal et al. 2019; Jacobson 2022). These pathogens can induce severe damage to the intestinal mucosa of piglets, leading to malabsorption, fluid and electrolyte imbalances, dehydration, and, frequently, mortality (Bergeland and Henry 1982).

Piglets are born agammaglobulinaemic, meaning they lack circulating antibodies, and their gastrointestinal tract is initially sterile. The passive immunity they acquire from the sow’s colostrum is therefore indispensable for their protection against early microbial threats (Rooke and Bland 2002; Le Dividich et al. 2005). Consequently, strategies to enhance the sow’s immunity to relevant pathogens are central to preventing neonatal diarrhoea (Krishna et al. 2020; Jacobson 2022). One possible strategy is “controlled oral exposure”, also known as ”feedback”. This protocol has been used in the swine industry for decades (Kohler 1974; Schwartz et al. 2013) and involves the intentional exposure of pregnant sows to farm-specific pathogens to elicit an immune response, thereby enhancing the transfer of specific antibodies to piglets via colostrum and milk. This approach is particularly valuable for diseases for which no effective commercial vaccines are available (Arruda 2010). Historically, feedback protocols involved administering faeces from diarrhoeic piglets or the ground intestines of dead piglets to sows (Arruda 2010; Schwartz et al. 2013). While this method directly exposes sows to the causative agents, including viruses, it also presents the risk of uncontrolled pathogen exposure (Arruda 2010). Another approach to feedback involves the formulation of “cocktails”, in which biological materials from diarrhoeic piglets are inoculated into a medium, such as cow’s milk, before being fed to sows (Neumann et al. 2020). This approach is expected to provide a safer way to expose sows to bacterial pathogens. Although fermented milk cocktails are sometimes used in practice, there is insufficient literature documenting their use. There are no studies examining the bacterial composition of fermented cocktails in relation to the inoculum used – the faeces of piglets with diarrhoea.

The objective of this study, therefore, was to analyse the bacterial composition of fermented milk used for sow feedback, to compare the bacterial composition of the fermentate with the initial faecal inoculum, and to assess the safety of its administration to pregnant sows.

## MATERIAL AND METHODS

### Faecal samples and milk fermentation

Pairs of faeces–fermented milk were obtained from a pig farm where feedback in the form of fermented milk inoculated with faeces from diarrhoea affected piglets had been utilised for approximately 10 years. Following the routine preparation, the faeces of diarrhoeic piglets were collected from various pens, mixed and used to inoculate five litres of commercially available longlife UHT cow’s milk. The fermentation process was carried out in 5 litre plastic bottles that were hermetically sealed and maintained at a temperature of 37 °C for 24 hours. A total of 16 pairs of faeces–fermented milk samples were collected.

### DNA isolation from stool and fermented milk

DNA from faeces and fermented milk was isolated using a QIAamp PowerFaecal Pro DNA Kit (Qiagen, Hilden, Germany) according to the manufacturer’s instructions. For each sample, 250 mg of stool or 250 μl of fermented milk sample was combined with lysis buffer and mechanical disruption beads, then homogenised. The concentration of the extracted DNA was quantified using a DeNovix DS11 FX spectrophotometer (DeNovix Inc., Wilmington, USA).

### Bacterial culture of *Clostridium perfringens*

*C. perfringens* was grown using Columbia blood agar containing 5% sheep blood (Labmediaservis, Jaroměř, Czech Republic) under anaerobic conditions (AnaeroGen; Oxoid, Basingstoke, UK). Semiquantification of *C. perfringens* was evaluated using a cross-scale (ranging from one cross for sparse growth to five crosses for abundant growth). The identity of selected colonies was confirmed using the MALDI Biotyper (Bruker Microflex mass spectrometer equipped with MALDI Biotyper Compass 4.1.100.10 software, Database RUO v12.0, updated version; Bruker, Billerica, MA, USA). To determine potential toxin production and to characterise the type of *C. perfringens*, toxin gene detection (*cpa*, *cpb*, *cpe*, *etx*, *iap*, *cpb2*) was performed using PCR (Baums et al. 2004). To test the impact of pH on *C. perfringens* growth, 100 μl of overnight cultures of three different *C. perfringens* strains in Wilkins–Chalgren broth (WCHB) (Thermo Scientific, Waltham, USA) were inoculated into 900 μl of WCHB adjusted to pH 5.0, 4.6, 4.2 or 3.8 using lactic acid. The cultures were then incubated under anaerobic conditions for 1, 3, and 6 h, after which bacterial growth was determined by plating serial dilutions on Columbia blood agar and counting colonies after 24 h of anaerobic culture.

### Microbiota composition determined by 16S rRNA gene sequencing

Microbiota composition was analysed by sequencing the V3–V4 variable region of the 16S rRNA gene using an external service (Eurofins, Luxembourg). The obtained raw data were processed with QIIME 2 (Bolyen et al. 2019) using built-in modules DADA2 (Callahan et al. 2016) for trimming and denoising demultiplexed sequences; taxonomic classification was performed using the Greengenes 2 database, v2022.10 (McDonald et al. 2022); sequence diversity was evaluated with diversity core-metrix-phylogenetic and differential abundance was analysed using the composition ANCOMBC module (Lin and Peddada 2020).

### The presence of alpha-toxin in fermented milk

The presence of alpha-toxin, a potential product of *C. perfringens*, in fermented milk was determined by the Monoscreen AgELISA *Clostridium perfringens* alpha-toxin (BioX Diagnostics S.A., Rochefort, Belgium) according to the manufacturer’s instructions.

### Statistical analysis

Significance of differences between samples was evaluated using the Kruskal–Wallis test (richness and evenness), PERMANOVA (beta diversity) and Principal Coordinate Analysis (PCoA) within QIIME 2 plugins. The Chisquared test was used to analyse the correlation between the presence or absence of two different bacterial taxa within samples. *P* < 0.05 was considered significant.

## RESULTS

### Diversity of faecal and fermented milk samples

**Figure 1:**
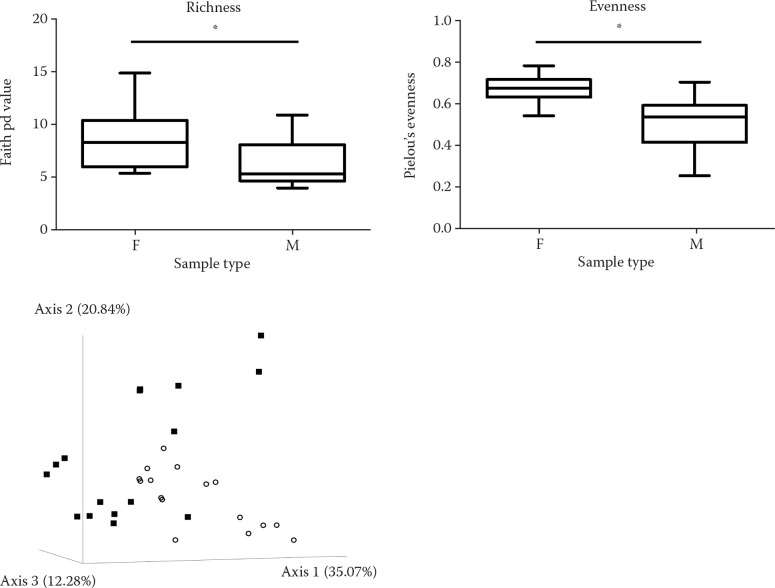
Diversity analysis of faecal and fermented milk samples (A) Richness of bacterial composition in faeces and in fermented milk. (B) Evenness of bacterial composition in faeces and in fermented milk. Asterisk indicate significant difference; The data are presented as maximum and minimum, as well as the median. The box represent the 25^th^ and 75^th^ percentiles of values. (C) PCoA analysis of beta diversity of faecal and fermented milk samples; The rings represent faecal samples, and the squares represent fermented milk samples F = faecal samples; M = fermented milk samples; PCoA = principal coordinate analysis; pd = phylogenetic diversity

Bacterial richness was significantly higher in faecal samples than in fermented milk (Figure 1A). Similarly, the evenness of bacterial species distribution was significantly higher in faecal than in fermented milk samples (Figure 1B). Analysis of beta diversity using PCoA also significantly separated faecal samples from fermented milk, showing that, despite using faecal samples as inoculum, specific conditions present in milk considerably diverted microbiota development during fermentation from the original inoculum (Figure 1C).

### Identification of bacterial species in the faeces of piglets with diarrhoea and in fermented milk samples

**Figure 2:**
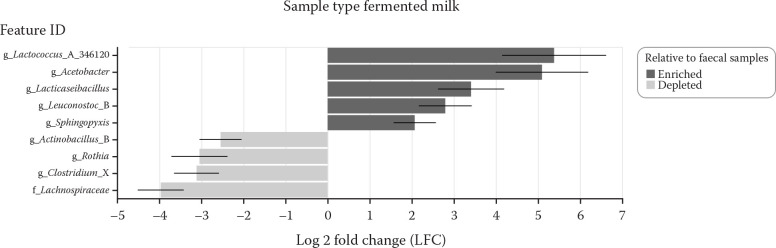
Bacterial genera and families differentially abundant in fermented milk samples in comparison to faecal inoculum The LFC is the estimated mean value of change in the log 2 abundance of a bacterial taxon in fermented milk samples when compared to faecal samples; The error bars represent the standard error of the mean

In total, 1 235 amplicon sequence variants (ASVs) were identified across all analysed samples. A significantly higher abundance of lactic acid bacteria was present in fermented milk samples (Figure 2) than in faecal inoculum, but bacteria metabolising polysaccharides were depleted in milk fermentates. Genera *Acetobacter* and *Lactococcus* were identified nearly exclusively in fermented milk samples. Different *Lactobacillus* species were found in both milk fermentates and faeces.

### The correlation of *C. perfringens* with other bacteria

**Figure 3:**
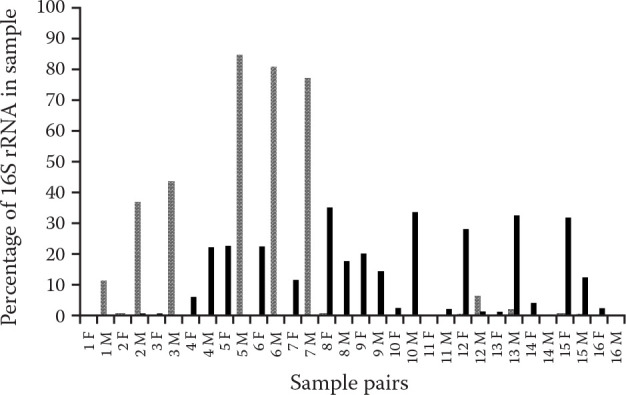
The correlation of *C. perfringens* and *Lactococcus lactis* A346120 The black columns represent the proportion of *C. perfringens* 16S rRNA out of all bacteria present; The grey pattern columns represent the proportion of *Lactococcus lactis* A346120; Numbers represent each pair of faecal and fermented milk pair of sample F = faecal samples; M = fermented milk samples

16S rRNA sequencing indicated that *C. perfringens*, a significant pathogen of the pig digestive tract, might be present in fermented milk samples. *C. perfringens* accounted for up to 33% of all bacteria in some fermentates (Figure 3), but not in others, even where the amount of *C. perfringens* in the faecal inoculum was high. The subsequent phase of the study therefore involved investigating the correlation between the presence of *C.* *perfringens* and that of other bacterial species in fermented milk samples. The only, but strong, negative correlation (chi-squared α = 5.02^–9^) was recorded for the presence of *C. perfringens* and *Lactococcus lactis* A346120 (Figure 3). When *Lactococcus lactis* A346120 reached 5% or more, *C. perfringens* did not exceed 5%, although in faecal samples that were used as an inoculum for milk fermentation, *C. perfringens* was present up to 22% (Samples 5F and 6F, Figure 3).

### Presence of *C. perfringens* in fermented milk samples

Since DNA sequencing can easily detect DNA from non-viable bacterial cells, 16S microbiome profiling can be misleading regarding the presence of viable bacteria in a sample. Therefore, the viability of *C. perfringens* was assessed by performing a semiquantitative culture detection of *C. perfringens* in faecal material and fermented milk samples. As the samples were collected in 2023 and 2024, only faecal and fermented milk samples that had been frozen at –20 °C for no longer than six months were evaluated for *C. perfringens* viability by semiquantitative culture. Viable *C. perfringens* was detected in all nine tested faecal samples, though in varying abundance, and in eight out of nine tested fermented milk samples (Table 1). *C. perfringens* was present in low abundance in samples with a pH below 4.88. However, when the pH did not decrease below this value, *C. perfringens* was highly abundant in three out of four samples (see Table 1).

**Table 1:** Estimation of *C. perfringens* viability using semiquantitative culture method and assessment of *C. perfringens* proportion in samples by 16S rRNA gene sequencing

Faecal sample	Faecal culture*	% of *C. perfringens* DNA in faeces**	Fermented milk sample	Fermented milk culture*	% of *C. perfringens* DNA in fermented milk**	pH of fermented milk
8 F	++++	34.892	8 M	+	17.581	4.23
9 F	+++	19.839	9 M	+	14.275	4.32
10 F	+	2.255	10 M	+	33.373	4.16
11 F	+	0.146	11 M	–	1.930	4.24
12 F	++++	37.833	12 M	+	1.117	3.98
13 F	+++++	1.024	13 M	++++	32.298	5.62
14 F	+++++	3.909	14 M	+	0.150	6.71
15 F	++++	31.593	15 M	++	12.259	4.88
16 F	+++++	2.183	16 M	++++	0	5.92

*Crosses represents the amount of *C. perfringens*, ranging from one cross for sparse growth to five crosses for the most extensive growth of* C. perfringens*; **Percentage determined by 16S rRNA gene sequencing

### Survival of *C. perfringens* under different pH conditions

**Figure 4:**
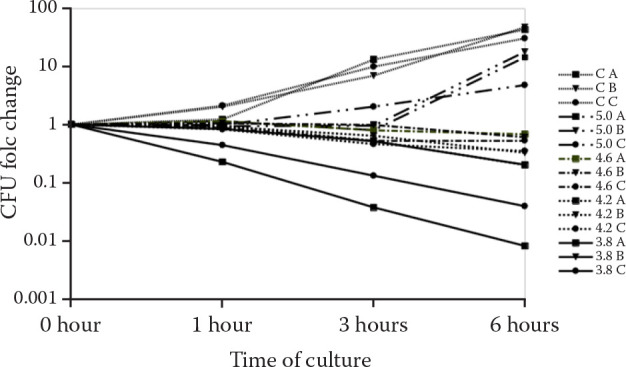
Survival of *C. perfringens* under different pH conditions The values represent the percentage of *C. perfringens* relative to the initial dose of inoculum at time points of 1, 3 and 6 h of cultivation. (A–C) Three different *C. perfringens* strains originating from piglet diarrhoea 5.0 = pH 5.0; 4.6 = pH 4.6; 4.2 = pH 4.2; 3.8 = pH 3.8

We further tested the hypothesis that *C. perfringens* growth is inhibited after a specific growth phase by a decrease in pH resulting from the fermentation of sugars by lactic acid bacteria, and that a further reduction in pH leads to partial inactivation. The results (Figure 4) show that pH 4.6 or lower inhibits the growth of *C. perfringens*. This finding corresponds with the results of semiquantitative cultivation of *C. perfringens* from fermented milk samples. In fermented milk samples with a pH below 4.8, the number of *C.* *perfringens* colonies was low (Table 1).

### Presence of *C. perfringens* alpha-toxin in fermented milk samples

Given *C. perfringens*’s ability to grow in milk, we further tested for the presence of *C. perfringens* alpha-toxin in milk fermentates. The alpha-toxin was not detected in any of the 16 tested fermented milk samples using the ELISA method. However, all *C.* *perfringens* isolates were positive for the alpha-toxin gene (*cpa*) and the beta2-toxin gene (*cpb2*). Still, it was negative for the beta-toxin gene (*cpb*), epsilon-toxin gene (*etx*), iota-toxin gene (*iap*), and enterotoxin gene (*cpe*). We thus conclude that the isolates were *C. perfringens* type A isolates typically present in piglet diarrhoeal faeces.

## DISCUSSION

The controlled oral exposure of sows is used with the intention of protecting suckling piglets against pathogens causing neonatal diarrhoea (Kohler 1974). The most commonly used and welldocumented method of controlled oral exposure is feeding sows the faeces of diseased piglets (Schwartz et al. 2013*)*. An alternative protocol involves feeding sows ground intestines from dead piglets. Although these protocols are effective in controlling diarrhoea caused by bacterial and viral pathogens, the primary concern is uncontrolled exposure of pregnant sows to these pathogens (Arruda 2010). Another approach is to provide sow’s milk fermented with faeces from diseased piglets (Neumann et al. 2020). Although uncertainty regarding microbial composition remains, it is reduced to bacteria capable of aerobic or semianaerobic growth in milk. In agreement with such expectations, microbiota richness and evenness were lower in fermented milk samples than in the original faecal samples, documenting the selective conditions during milk fermentation (Lichtenegger et al. 2024). Bacteria underrepresented or completely depleted in fermented milk samples were those metabolising polysaccharides (Flint et al. 2008). On the other hand, the prevailing bacteria, such as *Lactobacillus*, *Acetobacter*, and *Lactococcus*, are taxa previously reported in fermented milk (Zhong et al. 2016). Some of them (*Lactococcus*) also overlap with the microbiota found in fermented cheese (Korena et al. 2023), which is not too surprising, given that the same milk serves as the environment for microbial multiplication.

Interestingly, in some fermented milk samples, we found a high proportion of *C. perfringens* 16S rDNA. The high amount of *C. perfringens* 16S rDNA in fermented milk corresponded with a high amount of *C. perfringens* 16S rDNA in the faeces used as the inoculum. However, this was not the case in all pairs of faecal inoculum–fermented milk. In some pairs, the amount of *C. perfringens* 16S rDNA was high in faeces but low or not detected in fermented milk. Interestingly, the depletion of *C. perfringens* 16S rDNA was correlated with the presence of *Lactococcus lactis* A346120 in fermented milk. The presence of a high amount of viable *C. perfringens* in fermented milk may represent a potential health risk. Although production of alpha-toxin by *C. perfringens* has been associated with diarrhoea in piglets (Czanderlova et al. 2006), this toxin was not detected in any of the fermented milk samples. Milk as a substrate and the culture conditions used, thus, probably do not induce alpha-toxin expression. While this increases the safety of milk fermentates for sows, it also eliminates the chance of developing an antibody response to alpha-toxin (Salvarani et al. 2013). We further evaluated the viability of *C. perfringens* in fermented milk and found that it was dependent on the pH of the resulting fermented milk. In laboratory cultures, the viability of *C. perfringens* decreased when the pH was below 4.6. Similarly, the viability of *C. perfringens* decreased when the pH of fermented milk fell below 4.8. To increase the safety of milk fermentates, it might be advisable to check the pH of milk fermentates on farms and use only batches with pH lower than 4.6. In addition, since there are some species with negative correlation to *C. perfringens*, e.g. *Lactococcus lactis* A346120, it might be possible to obtain such strains in pure culture and add them in later phase of milk cultivation or together with faecal inoculum. Such approaches and control measures may improve the preparation of antigenrich but safe fermented milk feedback on farms.
